# Optimizing Radiation Risk Assessment in CT Imaging: Establishing Institutional Diagnostic Reference Levels and Personalized Dose Strategies for Chest, Abdomen, and Pelvis Scans

**DOI:** 10.3390/tomography11060065

**Published:** 2025-06-03

**Authors:** Zuhal Y. Hamd, Huda I. Almohammed, Elbagir Mansour, Abdoelrahman Hassan A. B., Awadia Gareeballah

**Affiliations:** 1 Department of Radiological Sciences, College of Health and Rehabilitation Sciences, Princess Nourah bint Abdulrahman University, P.O. Box 84428, Riyadh 11671, Saudi Arabia; zyhamd@pnu.edu.sa; 2 Department of Applied Radiologic Technology, College of Applied Medical Sciences, University of Jeddah, Jeddah 23218, Saudi Arabia; ahali@uj.edu.sa; 3 Department of Radiotherapy, College of Medical Radiologic Sciences, Sudan University of Science and Technology, Khartoum P.O. Box 11111, Sudan; abdoelrahmanhassan@sustech.edu; 4 Department of Diagnostic Radiology, College of Applied Medical Sciences, Taibah University, Al-Madinah Al-Munawwarah 42353, Saudi Arabia; agsali@taibahu.edu.sa

**Keywords:** computed tomography, radiation dose, dose optimization, diagnostic reference level, radiation protection, cancer risk

## Abstract

**Background:** As a diagnostic radiology procedure, computed tomography (CT) contributes to patient radiation exposure; hence, it deserves special consideration. The use of diagnostic reference levels (DRLs) is an efficient way to optimize patient radiation dosage. The computed tomography dose index volume (CTDIv) and the dose-length product (DLP) help to measure DRLs. **Methods:** A retrospective analysis was conducted on 106 patients (43.9% male, 56.1% female; mean age of 48.18 years) who underwent computed tomography chest, abdomen, and pelvis (CT CAP) scans using a Toshiba Aquilion Prime 160-slice CT scanner. Data included patient demographics, CT parameters (mA, tube rotation time, pitch, slice thickness, and slice count), and dose indices: dose length product (DLP), computed tomography dose index volume (CTDIvol), and effective dose. Cancer risks were calculated based on effective dose, patient demographics, and scan parameters. **Results:** This study demonstrated that the mean values for DLP, CTDIvol, and effective dose were 1719.64 ± 488.45 mGy·cm, 25.97 ± 6.96 mGy, and 27.5 ± 7.82 mSv, respectively. Cancer risk estimates ranged from 0.048% to 1.58%, with higher risks observed for females, younger patients. Significant correlations were found between dose indices and technical parameters, including pitch, kVp, tube rotation time, and slice thickness (*p* < 0.005). **Conclusions:** The mean values for DLP, CTDIvol, and effective dose for abdominopelvic scans were higher than those found in previous studies, with significant correlation of weight on these values. Optimizing CT protocols and establishing DRLs tailored to clinical indications are critical for minimizing radiation exposure and enhancing patient safety.

## 1. Introduction

Computed tomography (CT) imaging is a cornerstone of modern diagnostic medicine, providing critical insights into conditions affecting the chest, abdomen, and pelvis. However, its widespread use raises significant concerns about radiation exposure, particularly for vulnerable populations such as children and patients undergoing repeated scans. Optimizing radiation doses is essential to balancing diagnostic efficacy with patient safety, and this goal hinges on establishing Diagnostic Reference Levels (DRLs), personalized dose strategies, and technological advancements [1,2,3].

DRLs are benchmarks for safe imaging practices, allowing facilities to compare their dose levels against established standards to ensure patient safety and diagnostic quality. International bodies such as the International Commission on Radiological Protection (ICRP) and the International Atomic Energy Agency (IAEA) underscore the importance of DRLs in optimizing doses [4,5]. However, establishing universally accepted DRLs is complex, given the variability in scanner models, imaging protocols, and patient demographics. Significant variations in DRLs exist globally and between facilities, driven by differences in clinical practices and technological capabilities. These discrepancies highlight the need for research to harmonize DRLs while maintaining flexibility for regional and institutional adaptations [1,6].

Personalized dose strategies have emerged as a vital approach to address these challenges. By tailoring radiation doses to individual patient characteristics such as age, gender, and body composition, personalized protocols ensure diagnostic-quality images while minimizing exposure. For instance, McCollough et al. demonstrated that children and smaller patients require lower radiation doses than adults [7]. Quantifying radiation risks also plays a pivotal role in dose optimization. Laurier D et al. introduced the linear no-threshold (LNT) model to estimate cancer risks from ionizing radiation, emphasizing the need to minimize unnecessary exposure in line with ALARA (As Low As Reasonably Achievable) principles [8].

CT scans of the chest, abdomen, and pelvis are among the most common diagnostic imaging procedures but account for significant radiation exposure. Establishing DRLs for these scans is crucial in optimizing doses and ensuring patient safety. DRLs act as a reference point, enabling facilities to identify outliers and adjust practices accordingly. They align with the ALARA principle, aiming to minimize exposure while maintaining diagnostic accuracy [9,10].

The ICRP and IAEA provide comprehensive guidelines for defining DRLs, emphasizing their role in standardizing practices across facilities. The IAEA advocates for the regular update of DRLs and the sharing of international data to improve radiation safety worldwide. ICRP integrates DRLs into a broader framework of radiation protection, focusing on optimization, justification, and dose limitation. Both organizations stress the need to account for variations in patient demographics, scanner technologies, and imaging protocols when establishing DRLs, ensuring that benchmarks are locally relevant and globally consistent [5,9,11,12].

Optimizing radiation doses in CT imaging of the chest, abdomen, and pelvis is essential to balancing diagnostic accuracy with patient safety. The establishment of DRLs is a cornerstone of this optimization process, offering a standardized approach to dose management while accommodating regional and institutional variations. Personalized dose strategies, informed by patient-specific factors and supported by technological advancements, further refine this approach. Efforts to harmonize DRLs globally, as advocated by the ICRP and IAEA, and initiatives like those in Saudi Arabia demonstrate the potential for improved safety and efficacy in CT imaging practices. By prioritizing these strategies, healthcare providers can enhance patient care while mitigating the risks associated with ionizing radiation [4,13,14].

This study focused on CAP CT scans as they are widely used in clinical practice, particularly for oncological evaluation (the sample of this study comprised cancer patients being screened for staging and follow-up). Subsequent CAP examinations cover multiple anatomical regions in a single scan. They contribute significantly to cumulative radiation exposure, making it crucial to establish appropriate DRLs for optimizing radiation dose while maintaining diagnostic accuracy.

## 2. Materials and Methods

This retrospective cross-sectional study assessed radiation dosage associated with CT chest, abdomen, and pelvis (CAP). In total, 106 adult patients were included in the study with the following indications: cancer characterization, staging, and follow-up. Data were collected over three months in 2023 from three scanners manufactured by the same company. Patient demographic data, including age, gender, weight, height, BMI, and clinical indications, were recorded. Scanning parameters included mA per rotation, number of slices, pitch, kVp, gantry rotation time, slice thickness, and field of view (FOV).

### 2.1. CT Machines

The scans were performed using a Toshiba Aquilion Prime 160-slice CT scanner (TSX-303A) (Toshiba Medical Systems, Otawara, Japan, 2016). This system employed adaptive iterative dose reduction techniques for optimized low-dose imaging through iterative reconstruction. Routine quality control measures were performed on the CT machine to ensure consistency in radiation output, dose delivery, and protocol parameters.

### 2.2. Radiation Dosimetry

The effective dose (ED) was estimated using region-specific conversion factors (k) applied to the dose-length product (DLP), following ICRP 103 guidelines. For chest–abdomen–pelvis CT scans, a k-value of approximately 0.015 mSv/mGy·cm was used. This method provides an approximate estimation of stochastic radiation risk when direct organ dose measurements are not available.

Radiation dose parameters, including CTDIvol and DLP, were directly extracted from the CT scanner’s dose report for each examination. These values were automatically generated by the Toshiba Aquilion Prime CT system during the scanning process, ensuring accurate and reliable measurement of radiation exposure. The effective dose was calculated using the following formula:

(1) $$\mathrm{Effective}\;\mathrm{Dose}\;(\mathrm{mSv})\;\mathrm{ED}=\Sigma_{T}\omega_{T}H_{T}$$

where *H_T_* is the equivalent dose in organ or tissue *T*, and *w_T_* is the weighting factor for tissue *T* [15].

According to ICRP publication 119 [15], the equivalent dose, H_T,R_, in tissue or organ T due to radiation R, is given by H_T,R_ = w_R_D_T,R_, where D_T,R_ is the average absorbed dose from radiation R in tissue T and w_R_ is the radiation weighting factor. Since w_R_ is dimensionless, the units are the same as for absorbed dose (J/kg), and its special name is sievert (Sv). The total equivalent dose H_T_ is the sum of H_T,R_ over all radiation types: H_T_ = ∑ _R_ H_T;R_.

According to ICRP 60 (ICRP 1991), the effective dose is defined as the weighted average of organ dose values HT for several specified organs. How much a particular organ contributes to calculating effective dose depends on its relative sensitivity to radiation-induced effects, as represented by the tissue-weighting factor w*_i_* attributed to the organ [16].

### 2.3. CT CAP Examination Protocols

The scan range extended from the lung apices to the lesser trochanter, with patients positioned supine, feet first, and arms raised above their heads. Scout images in anteroposterior and lateral projections were acquired to determine the area of interest and ensure precise positioning. Tube current modulation was applied to distribute the dose evenly across the trunk, with Auto mA adjusted based on patient size and scan requirements. The kVp was set at 120 for standard scans, with 140 kVp used selectively. Patients were instructed to hold their breath to minimize motion artifacts. Scan phases were tailored to clinical indications, with scout, plain, and portal venous phases commonly used. Additional phases, such as arterial or delayed, were performed for specific cases, such as liver metastases or renal issues, with the scan area limited to the upper abdomen or as required.

### 2.4. Data Analysis

Patient demographics, scanning parameters, CTDIvol, DLP, and effective dose data were extracted in Microsoft Excel for window 10; then, the data were analyzed using SPSS version 27 (IBM, Armonk, NY, USA) and the DATAtab Online Statistics Calculator (DATAtab e.U. Graz, Austria) and Jamovi software 2.4.11. Inferential statistics were used with minimum, mean, and standard deviation values, which were calculated for continuous data; then, regression analysis, Pearson’s correlation, independent sample *t*-test, and one-way ANOVA test were used to assess the correlations between dose indices, patient demographic data, and scan parameters, with a *p* value of <0.01 and <0.05 considered statistically significant.

### 2.5. Ethical Approval

The study was approved by the institutional review board at Princess Nourah bint Abdulrahman University. IRB Log Number: 23-0206.

## 3. Results

Table 1 provides an outline of the demographic characteristics of the study participants. In terms of gender, more than half were females at 55.7%, and 44.3% were males. When classified by age, the majority (72.6%) were in the age range of 19–59 years. Regarding height, 20.8% were in the height range between 140 and 155 cm, 56.6% were between 156 and 170 cm, and 22.6% were between 171 and 185 cm. Lastly, for weight distribution, the majority (58.5%) were within 61–85 kg.

The findings revealed that the average weight, height, and BMI were 73.39 kg ± 15.16, 1.61 m ± 0.095, and 26.2 kg/m^2^ ± 0.31, respectively. The values for KVP (kilovolt peak), mA per R (milliampere per rotation), duration, slice thickness, and the number of slices were 120.57 ± 0.33, 472.42 ± 158.32, 0.61 ± 0.11, 3.38 mm ± 0.37, and 189.09 ± 27.28, respectively. The pitch parameter ranged from 0.50 to 1.37, with a mean of 0.74 ± 0.27. The field of view (FOV) remained constant at 330 cm. The CTDI_vol_ ranged from 9.42 to 39.54, with an average value of 25.96 ± 6.96. The dose-length product (DLP) varied from 587.00 to 2752.48, with a mean of 1719.63 ± 488.45, whereas the effective dosage ranged from 9.39 to 44.04, with a mean of 27.51 ± 0.81 (Table 2).

Table 3 presents the correlation of demographic and exposure parameters in both sexes. For weight, males had a higher mean weight of 74.04 ± 15.73 kg, while females were 72.86 ± 14.80 kg, with no significant difference (*p* = 0.695; 95% CI: −4.770 to 7.126). Height and BMI were significantly higher in males compared to females, at 1.69 ± 0.07 m compared to 1.56 ± 0.067 m for females (*p* < 0.001; 95% CI: 0.10514 to 0.15867) and 2.86 ± 0.24 kg/m^2^ compared to 2.43 ± 0.21 kg/m^2^ (*p* < 0.001 and a 95% CI of 0.34085 to 0.51651), respectively. Concerning the differences in exposure parameters used in both genders, the results demonstrated that they insignificantly differed between males and females; the mean KVP values were similar between males (120.43 ± 2.92) and females (120.68 ± 3.65), with no significant difference (*p* = 0.693; 95% CI: −1.517 to 1.013). The mA parameter showed slightly higher values for females (486.25 ± 169.29) compared to males (455.06 ± 143.36, *p* = 0.307; 95% CI: −91.429 to 29.048). Rotation time was nearly identical between males (0.60 ± 0.10) and females (0.61 ± 0.11, *p* = 0.503; 95% CI: −0.0553 to 0.0273). The mean slice thickness was 3.37 ± 0.38 mm for males and 3.39 ± 0.38 mm for females (*p* = 0.802; 95% CI: −0.16496 to 0.12781). However, the number of slices differed significantly, with males averaging 199.64 ± 29.08 compared to 180.69 ± 22.71 for females (*p* < 0.001; 95% CI: 8.665 to 29.222). The pitch parameter was nearly symmetrical: 0.75 ± 0.29 for males and 0.74 ± 0.26 for females (*p* = 0.850; 95% CI: −0.09845 to 0.11931). The field of view (FOV) was constant at 330.00 ± 0.00 cm for both sexes.

The CTDI_vol_ parameter illustrated a significant difference between males and females, while the other dose parameters, including DLP, effective dose, and cancer risk, are statistically similar between sexes. The mean CTDIvol for males was 24.30 ± 6.81, which was significantly lower than that for females (27.30 ± 6.85), with a *p*-value of 0.027 and a 95% CI of −5.64991 to −0.35088. However, the DLP (dose-length product) values insignificantly differed, with males averaging 1695.18 ± 493.61 compared to 1739.12 ± 487.66 for females (*p* = 0.648; 95% CI: −234.43353 to 146.54707). The effective dose was also comparable between sexes, with the average for males being 27.121 ± 7.90 and for females being 27.83 ± 7.80 (*p*-value =0.648, 95% CI ranged from −3.75094 to 2.34475), indicating no significant difference. Similarly, the cancer risk insignificantly differed, with slightly higher values in females (0.54 ± 0.34) than in males (0.46 ± 0.28) (*p* = 0.184; 95% CI: −0.20208 to 0.03940) (Table 4).

**Figure 1:**
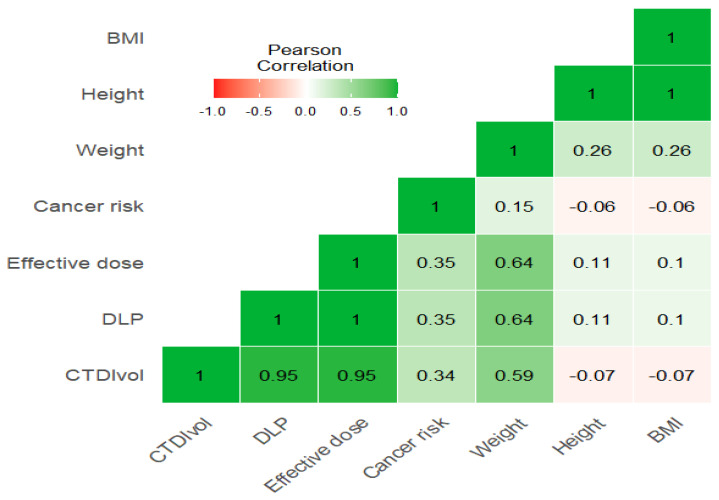
Heatmap for correlation between weight, height, BMI and CTD/vol, DLP and effective dose.

**Figure 2:**
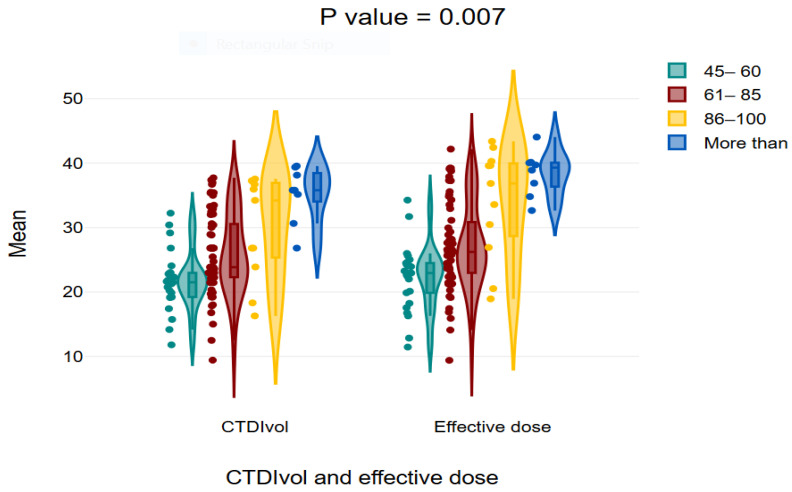
Violin plot showing the mean values of effective dose and CTDIvol in different weight categories. It shows that as the weight increase the values increase significantly.

The correlation heatmap in Figure 1 shows a moderate significant correlation between weight and CTDIvol, and a strong correlation between weight and DLP and effective dose (r = 0.59, 0.64, 0.64, respectively), while there is an insignificant impact of height and BMI with these exposure parameters (r = −0.07, 0.1, 0.1 for both height and weight with parameters). Furthermore, there is a strong significant correlation between CTDIvol with DLP and effective dose (r = 0.95) (Figure 1 and Figure 2)**.**

**Figure 3:**
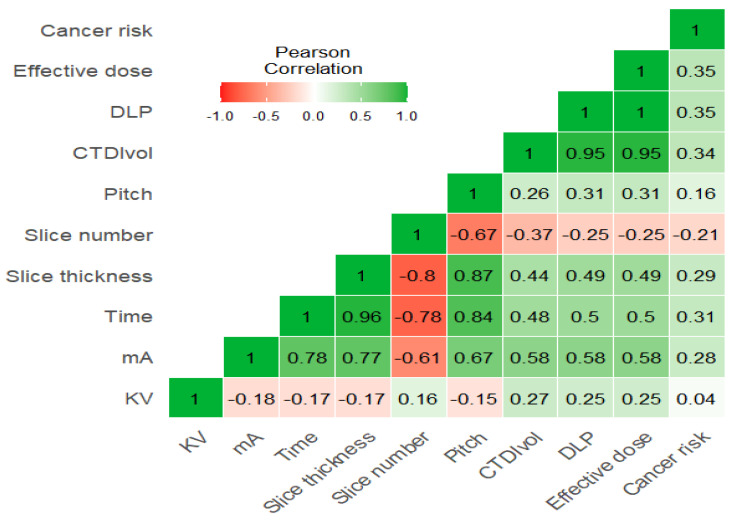
Correlation heatmap showing the relationship between exposure parameters and CTD/vol, DLP, and effective dose.

It was found that there were weak positive correlations of KVp with CTDIvol, DLP, and effective dose (r = 0.27, 0.25, 0.25), while there was no significant correlation between KVp and cancer risk (r = 0.04). Furthermore, MA is strongly correlated with time and pitch (r = 0.78, 0.67, respectively) and moderately correlated with CTDIvol, DLP, and effective dose (r = 0.58). Time demonstrates a strong correlation with pitch (r = 0.84), a moderate correlation with CTDIvol, DLP, and effective dose (r = 0.48, 0.5, 0.5, respectively), and a weak correlation with cancer risk (r = 0.31). Similarly, pitch shows weak correlations with CTDIvol, DLP, and effective dose (r = 0.26, 0.31, 0.31, respectively), while cancer risk is moderately correlated with CTDIvol, DLP, and effective dose (0.34, 0.35, 0.35, respectively) (Figure 3).

The findings indicate that the middle age group of 40–59 years received the highest mean values for CTDIvol, DLP, and effective dose, whereas the elderly group of 80–99 years received lower values, with statistically significant differences seen across the age groups. The mean CTDIvol values were 23.26 ± 6.16 for the age range of 19–39 years, 28.78 ± 7.34 for 40–59 years, 25.82 ± 5.86 for 60–79 years, and 21.00 ± 5.05 for 80–99 years (*p*-value = 0.002). The mean dose-length product (DLP) was 1548.76 ± 442.81 for 19–39 years, 1886.86 ± 516.37 for 40–59 years, 1716.49 ± 427.46 for 60–79 years, and 1748.5 ± 461.05 for 80–99 years (*p* = 0.015). The average effective values were 24.78 ± 7.08 for ages 19–39, 30.18 ± 8.26 for ages 40–59, 27.46 ± 6.83 for ages 60–79, and 23.65 ± 7.37 for ages 80–99, (Table 5).

The results of this study show that the dose parameters have no statistically significant differences between the two groups of BMI (*p* > 0.05) (Table 6).

**Figure 4:**
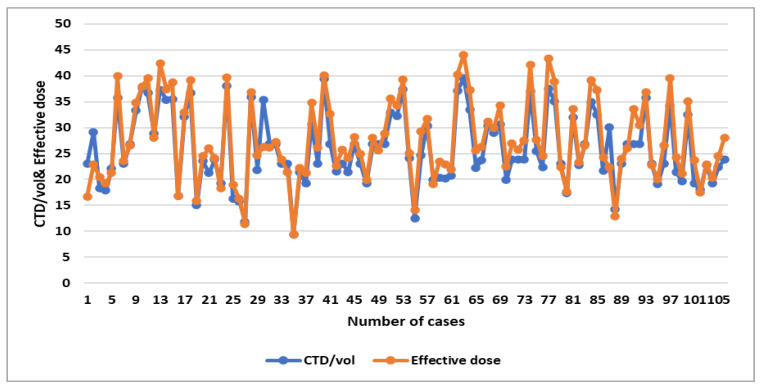
Line graph showing the relationship between effective dose and CTDIvol.

**Figure 5:**
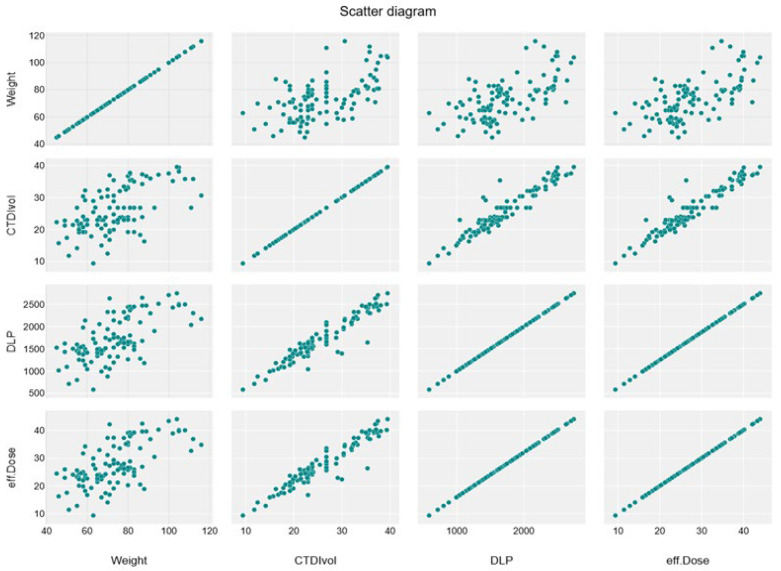
Scatter diagram shows positive linear association between weight and CTDIvol, DLP, and effective dose.

**Figure 6:**
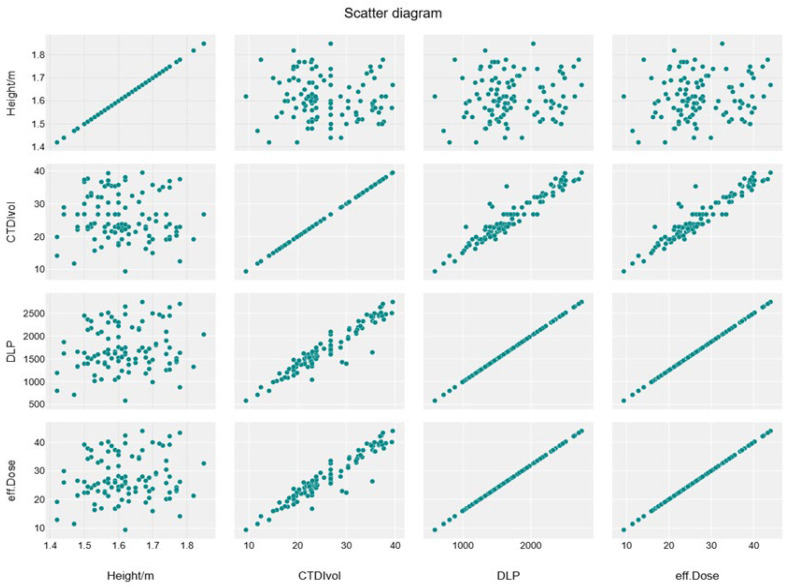
Scatter diagram showing no association between height and CTDIvol, DLP, and effective dose.

A significant moderate positive linear association was noticed between weight and CTDIvol, DLP, and effective dose (R^2^ = 0.3454, 0.4056, and 0.4056, respectively) (Table 7), which shows the relationship between dose parameters, as shown in the linear regression scatterplot and the line graph (Figure 3, Figure 4, Figure 5 and Figure 6).

In Table 8, it is demonstrated that the CT dose parameters in CAP in this study were higher than in the literature.

## 4. Discussion

Diagnostic reference levels (DRLs) are important methods for optimizing radiation dose in computed tomography (CT) examinations. Multiple studies have established DRLs for common CT procedures, including head, chest, and abdomen–pelvis scans, using CTDIvol and DLP as primary metrics [30,31,32,33]. This study assessed the radiation doses and associated cancer risks in CT chest, abdomen, and pelvis (CAP) procedures, focusing on the impact of scan parameters and patient demographics. The mean CTDIvol of 25.97 ± 6.96 mGy was higher than reported in similar studies, such as 22.94 mGy in Mansoor et al. and 10–12 mGy in Foley et al., likely due to increased scan length and a higher mean mA per rotation (472.42 mA). Tube current modulation effectively adjusted mA based on tissue thickness, but DLP values (587–2752.48 mGy·cm, mean 1719.64 ± 488.45 mGy·cm) remained relatively high, emphasizing the need to optimize the number of scanning phases based on clinical indications [2,3].

Cancer risk for trunk CT scans ranged from 0.048% to 1.581% (mean: 0.5%), with younger age groups (29–39 years) experiencing higher risks due to increased scan lengths and slice counts. In this study, it was found that as the weight of patients increased, the DLP and CTDIvol and effective dose significantly increased, while height and BMI produced an insignificant effect on these parameters. In contrast to this study, Sebelego et al. mention that patients with higher BMI also showed elevated DRL dose parameters [33].

Technical parameters such as rotation time, pitch, kVp, and slice thickness significantly influenced dose measures. Longer rotation times increased DLP, CTDIvol, and effective dose linearly. Higher kVp (140 vs. 120 kVp) and increased slice thickness (3.75 mm vs. 3.0 mm) also led to substantial dose increases, consistent with findings by Wang et al. Adjusting pitch values showed an expected reduction in radiation dose, with higher doses observed at 0.9 pitch than other values, aligning with previous studies [13].

In this study, it was found that the DLP (dose-length product) values insignificantly differed among both genders, with males averaging 1695.18 ± 493.61 compared to 1739.12 ± 487.66 for females (*p* = 0.648; 95% CI: −234.43353 to 146.54707). Alashban and Shubayr et al.’s results were consistent with this study; they found that the mean DLP values for CT were higher for females compared to male patients, with no statistically significant differences [17].

The study identified weak positive relationships between KVp and mA with CTDIvol, DLP, and effective dose, although no significant association was seen between KVp and cancer risk, suggesting that although KVp affects radiation dosage, its effects on cancer risk are negligible. This aligns with prior studies indicating that KVp exerts a relatively minimal impact on radiation dose and cancer risk, as it predominantly affects the energy of the X-ray beam rather than the dose itself. Time had a substantial link with pitch (r = 0.84) and a moderate correlation with radiation dose indices. Pitch demonstrated poor relationships with dose indices, suggesting that modifications to pitch have a limited impact on radiation exposure. The risk of cancer exhibited moderate relationships with CTDIvol (r = 0.34), DLP (r = 0.35), and effective dose (r = 0.35), hence affirming the anticipated association between elevated radiation exposures and heightened cancer risk. A previous study indicates that the radiation dose escalates linearly with rising mAs and exponentially with increasing kVp, irrespective of phantom size. Image noise diminished with reduced radiation doses but stabilized at elevated levels. Another study indicates that when the pitch value increases, the computed tomography dose index (CTDI) diminishes. Augmenting the pitch consequently leads to an extended scan duration. The radiation dosage observed in the phantom was consistent across all pitch options on the evaluated multislice helical CT equipment. The unforeseen outcome resulted from an automated proportional escalation in the tube current following an increase in pitch selection. The radiation dosage (CTDIvol and SSDE) escalated with phantom size at standard pitch; however, for elevated pitch, the dose increased up to a threshold and then declined linearly with increasing pitch [34,35,36,37].

Gender differences were evident, with females experiencing a higher mean cancer risk (0.539%) than males (0.457%), reinforcing findings from prior studies. Additionally, the number of scanning phases emerged as a critical factor, with triple-phase procedures nearly doubling cancer risk (0.37% for single-phase to 0.69% for triple-phase scans). These multiphase studies were often necessary for tumor staging and follow-up, particularly for organ-specific lesions [15].

The parameters of CT dose (CTDI_vol_, DLP, and effective dose) in this study were more than in other studies in different regions [18,19,20,21,22,23,24,25,26,27,28,29], which underscores the urgent need for optimized CT protocols to minimize radiation exposure while maintaining diagnostic accuracy. Implementing justification criteria for scan phases, employing advanced dose reduction technologies, and adhering to the ALARA principle (As Low as Reasonably Achievable) are essential. Future research should focus on developing diagnostic reference levels (DRLs) tailored to specific populations and clinical scenarios to enhance patient safety.

## 5. Conclusions

This study concludes that CT CAP procedures are associated with higher radiation doses, as evidenced by elevated CTDIvol, DLP, effective dose, and cancer risk compared to previous studies. These findings underscore the need for clear strategies to minimize radiation exposure. Cancer risk increases proportionally with higher CT doses and is notably higher in females and younger adults. To mitigate these risks, it is essential to ensure that higher doses are limited to cases where they are clinically necessary to balance diagnostic accuracy with patient safety.

### Limitations and Recommendations

This study faced several limitations. It was a mono-center study, with a smaller sample size of only one hundred and six, which may limit the generalizability of the results. Moreover, only one reconstruction method was applied, even though advanced iterative and deep learning-based reconstruction methods might affect image quality and dosage parameters. Future research should be conducted in multiple centers, employing several pieces of equipment in a larger patient cohort, and applying several reconstruction techniques to increase the robustness and pertinency of the conclusions. A key limitation of our dosimetric approach is the reliance on region-specific conversion factors (k) applied to DLP rather than direct organ dose (HT) measurements. While this method is practical and widely adopted, it does not account for patient-specific anatomical variations and may introduce uncertainty in individual risk estimation.

## Figures and Tables

**Table 1:** Demographic characteristics for the study participants.

Demographic Characteristics		Frequency	Percent
Gender	Male	47	44.3
	Female	59	55.7
Age groups	19–39	35	33.0
	40–59	42	39.6
	60–79	25	23.6
	80–99	4	3.8
Height/cm	140–155	22	20.8
	156–170	60	56.6
	171–185	24	22.6
Weight/kg	45–60	25	23.6
	61–85	62	58.5
	86–100	11	10.4
	>100	8	7.5
BMI/kg/cm^2^	≤25	39	27.12
	>25	67	25.29
Total		106	100

**Table 2:** Descriptive statistics for weight, height, BMI, CT parameters, and dose level.

Parameters	Minimum	Maximum	Mean	Std. Dev	Median
Weight/kg	45	116	73.39	15.16	73
Height/m	1.42	1.85	1.61	0.095	1.60
BMI/kg/m^2^	2.02	3.42	2.62	0.31	2.56
KVP	120	140	120.57	3.33	120
mA/R	175	699	472.42	158.32	484.50
Time/rots	0.5	0.9	0.61	0.11	0.70
Slice thickness/mm	3.00	3.75	3.38	0.37	3.50
Number of slices	120	256	189.09	27.28	184
Pitch	0.50	1.37	0.74	0.27	0.7
FOV (cm)	330	330	330.00	0.000	330
CTDI_vol_	9.42	39.54	25.96	6.964	23.85
DLP	587.00	2752.48	1719.63	488.45	1622.81
Effective dose	9.39	44.04	27.51	7.81	25.96
Cancer risk	0.04	1.58	0.50	0.31	0.41

**Table 3:** Comparison of mean height, weight, BMI, and CT exposure parameters in both genders.

Demographic and Exposure Parameters	Sex	N	Mean ± Std. Dev	*p*-Value	95% CI	
Weight/kg	Male	47	74.04 ± 15.73	0.695	−4.770	7.126
	Female	59	72.86 ± 14.80			
Height/m	Male	47	1.69 ± 0.07	<0.001	0.10514	0.15867
	Female	59	1.56 ± 0.067			
BMI/Kg	Male	47	2.86 ± 0.24	<0.001	0.34085	0.51651
	Female	59	2.43 ± 0.21			
KVP	Male	47	120.43 ± 2.92	0.693	−1.517	1.013
	Female	59	120.68 ± 3.65			
mA per R	Male	47	455.06 ± 143.36	0.307	−91.429	29.048
	Female	59	486.25 ± 169.29			
Time/rots	Male	47	0.60 ± 0.10	0.503	−0.0553	0.0273
	Female	59	0.61 ± 0.11			
Slice thickness/mm	Male	47	3.37 ± 0.38	0.802	−0.16496	0.12781
	Female	59	3.39 ± 0.38			
Slice number	Male	47	199.64 ± 29.08	<0.001	8.665	29.222
	Female	59	180.69 ± 22.71			
Pitch	Male	47	0.75 ± 0.29	0.850	−0.09845	0.11931
	Female	59	0.74 ± 0.26			
FOV (cm)	Male	47	330.00 ± 0.00	1.000		
	Female	59	330.00 ± 0.00			

**Table 4:** Comparison of mean CTDIvol, DLP, effective dose, and cancer risk among both genders.

Dose Parameters	Sex	Mean ± Std. Dev	*p*-Value	95% CI	
CTDIvol	Male	24.30 ± 6.81	0.027	−5.64991	−0.35088
	Female	27.30 ± 6.85			
DLP	Male	1695.18 ± 493.61	0.648	−234.43353	146.54707
	Female	1739.12 ± 487.66			
Effective Dose	Male	27.121 ± 7.90	0.648	−3.75093647	2.34475308
	Female	27.83 ± 7.80			
Cancer Risk	Male	0.46 ± 0.28	0.184	−0.2020752	0.0394001
	Female	0.54 ± 0.34			

**Table 5:** Comparison of mean CTDIvol, DLP, and effective dose in different age groups.

Dose Measurements	Age Groups/Years				*p*-Value
		40–59	60–79	80–99	
CTDIvol	23.26 ± 6.16	28.78 ± 7.34	25.82 ± 5.86	21.00 ± 5.05	0.002
DLP	1548.76 ± 442.81	1886.86 ± 516.37	1716.49 ± 427.46	1748.5 ± 461.05	0.015
Eff. Dose	24.78 ± 7.08	30.18 ± 8.26	27.46 ± 6.83	23.65 ± 7.37	0.015
Cancer risk	0.61 ± 0.36	0.52 ± 0.28	0.38 ± 0.24	0.14 ± 0.09	0.004

**Table 6:** Comparison of mean CTDIvol, DLP, and effective dose with BMI.

Dose Parameters	BMI/Kg/m^2^	N	Mean	Std. Deviation	*p*-Value
CTDIvol	≤25	39	27.12	7.17	>0.05
	>25	67	25.29	6.80	
DLP	≤25	39	1698.16	493.70	
	>25	67	1732.13	488.67	
Eff. Dose	≤25	39	27.17	7.89	
	>25	67	27.71	7.81	

**Table 7:** Linear regression demonstrating the relationship between weight and dose parameters.

Dose Parameters		Unstandardized Coefficients		Standardized Coefficients	t	R^2^	*p*-Value
		B	Std. Error	Beta			
CTDIvol	(Constant)	6.152	2.731	0.588	2.253	0.345	<0.001
	Weight/kg	0.270	0.036		7.408		
DLP	(Constant)	213.523	182.506	0.637	1.170	0.406	<0.001
	Weight/kg	20.523	2.436		8.425		
Effective dose	(Constant)	3.416	2.920		1.170	0.406	<0.001
	Weight/kg	0.328	0.039	0.637	8.425		

**Table 8:** Comparison of the dose parameter measurements with some national and international studies.

Author(s)	Year	Region of Scan	Area	Mean or Median and Range			Cancer Risk
				CTDIvol (mGy)	DLP (mGy·cm)	Effective Dose (mSv)	
Foley [2]	2012	abdomen and pelvic	Ireland	12	600	-	-
Elnour et al. [9]	2021	CAP	Sudan	6	970	9.9	-
Alashban & Shubayr [17]	2022	CAP	Saudi Arabia	-	-	10.75	4.41 × 10^−4^
Alkhorayef, M. [18]	2018	CAP	Saudi Arabia	12.0 (8.1–17.0)	740 (400.7–1100.0)	11.8 (6.4–17.1)	1/1500
Qurashi et al. [19]	2014	CAP	Saudi Arabia	15	1000	-	-
Ait Ouaggou et al. [20]	2024	CAP	Morocco	12.9	849.3	-	-
Alenazi [21]	2024	CAP	Saudi Arabia (review)	12.00–22.94	740 to 1493.8	-	-
Alashban & Shubayr [22]	2022	CAP	Saudi Arabia	3.93–29.14	284.10–2216.70	-	-
Palorini et al. [23]	2013	CAP	Italian survey	17	1200	-	-
Radaideh et al. [24]	2023	CAP	Jordan	19.3	1152	17.25	-
Bouchareb et al. [25]	2023	CAP	Oman	20	807	18.1	-
Wambani, J.S. et al. [26]	2010	Abdomen and pelvic	Kenya (survey)	18 (9–24)	1182 (1450–1950)	18 (6.8–29.2)	-
Lee et al. [27]	2020	CAP	Australia	11	940	-	-
Stadnyk et al. [28]	2023	Abdomen and pelvic	Ukraine (survey)	22	845	-	-
EL Fahssi et al. [29]	2024	CAP (with contrast)	Morocco	8.51	571.30	7.13	
This study	2025	CAP	Saudi Arabia	25.96	1719.63	27.51	0.5 ± 0.31

## Data Availability

The from the corresponding author upon a reasonable request.
